# Cost–effectiveness of emergency care interventions in low and middle-income countries: a systematic review

**DOI:** 10.2471/BLT.19.241158

**Published:** 2020-02-25

**Authors:** Kalin Werner, Nicholas Risko, Taylor Burkholder, Kenneth Munge, Lee Wallis, Teri Reynolds

**Affiliations:** a Department of Surgery, Division of Emergency Medicine, F51-62, Old Main Building, Groote Schuur Hospital, Faculty of Health Sciences, University of Cape Town, Observatory, Cape Town, 7925, South Africa.; b Johns Hopkins University School of Medicine, Department of Emergency Medicine, Baltimore, United States of America (USA).; c Department of Emergency Medicine, University of Southern California, Los Angeles, USA.; d KEMRI-Wellcome Trust Research Programme, Kilifi, Kenya.; e Department for Clinical Services and Systems, Integrated Health Services, World Health Organization, Geneva, Switzerland.

## Abstract

**Objective:**

To systematically review and appraise the quality of cost–effectiveness analyses of emergency care interventions in low- and middle-income countries.

**Methods:**

Following the PRISMA guidelines, we systematically searched PubMed®, Scopus, EMBASE®, Cochrane Library and Web of Science for studies published before May 2019. Inclusion criteria were: (i) an original cost–effectiveness analysis of emergency care intervention or intervention package, and (ii) the analysis occurred in a low- and middle-income setting. To identify additional primary studies, we hand searched the reference lists of included studies. We used the Consolidated Health Economic Evaluation Reporting Standards guideline to appraise the quality of included studies.

**Results:**

Of the 1674 articles we identified, 35 articles met the inclusion criteria. We identified an additional four studies from the reference lists. We excluded many studies for being deemed costing assessments without an effectiveness analysis. Most included studies were single-intervention analyses. Emergency care interventions evaluated by included studies covered prehospital services, provider training, treatment interventions, emergency diagnostic tools and facilities and packages of care. The reporting quality of the studies varied.

**Conclusion:**

We found large gaps in the evidence surrounding the cost–effectiveness of emergency care interventions in low- and middle-income settings. Given the breadth of interventions currently in practice, many interventions remain unassessed, suggesting the need for future research to aid resource allocation decisions. In particular, packages of multiple interventions and system-level changes represent a priority area for future research.

## Résumé

**Objectif:**

Revoir systématiquement et évaluer la qualité des analyses de rentabilité des interventions d'urgence dans les pays à faible et moyen revenu.

**Méthodes:**

Conformément aux lignes directrices de la méthode PRISMA, nous avons effectué une recherche systématique des études publiées avant mai 2019 sur PubMed®, Scopus, EMBASE®, Cochrane Library et Web of Science. Les critères d'inclusion étaient les suivants : (i) analyse de rentabilité originale des interventions ou ensembles d'interventions d'urgence, et (ii) analyse menée dans des pays à faible et moyen revenu. Pour identifier d'autres recherches primaires, nous avons également dépouillé les listes de référence des études incluses. Enfin, nous avons appliqué la grille CHEERS (Consolidated Health Economic Evaluation Reporting Standards) pour évaluer la qualité des études prises en compte.

**Résultats:**

Sur les 1674 articles identifiés, 35 articles correspondaient aux critères d'inclusion. Nous avons également repéré quatre études supplémentaires dans les listes de référence. En revanche, nous avons exclu de nombreuses études car elles ne tenaient compte que des évaluations de coûts sans effectuer d'analyse de rentabilité. La plupart des études retenues étaient des analyses portant sur une seule intervention. Les interventions d'urgence examinées par ces études comprenaient les services préhospitaliers, la formation des prestataires, les programmes de traitement, les établissements et outils diagnostiques d'urgence, ainsi que le continuum de soins. La qualité des rapports figurant dans ces études était variable.

**Conclusion:**

Nous avons trouvé de vastes lacunes dans les indications relatives à la rentabilité des interventions d'urgence dans les pays à faible et moyen revenu. Étant donné la large gamme d'interventions pratiquées actuellement, nombre d'entre elles ne font encore l'objet d'aucune évaluation. Lors de futures recherches, il pourrait donc s'avérer nécessaire d'aider à la prise des décisions liées à l'attribution des ressources. Ces futures recherches devront être orientées en priorité vers les ensembles constitués de multiples interventions ainsi que vers les changements systémiques.

## Resumen

**Objetivo:**

Revisar y evaluar sistemáticamente la calidad de los análisis de rentabilidad de las intervenciones de atención de emergencia en los países de ingresos bajos y medios.

**Métodos:**

Siguiendo las directrices de PRISMA, se realizaron búsquedas sistemáticas en PubMed®, Scopus, EMBASE®, Cochrane Library y Web of Science sobre los estudios publicados antes de mayo de 2019. Los criterios de inclusión fueron: (i) un análisis original de rentabilidad de la intervención de atención de emergencia o del paquete de intervenciones, y (ii) el análisis se llevó a cabo en un lugar de ingresos bajos y medios. Para identificar estudios primarios adicionales, se realizaron búsquedas manuales en las listas de referencias de los estudios incluidos. Se utilizó la directriz de las Normas consolidadas de los informes de la evaluación económica de la salud (*Consolidated Health Economic Evaluation Reporting Standards*) para evaluar la calidad de los estudios incluidos.

**Resultados:**

De los 1674 artículos que se identificaron, 35 artículos cumplían los criterios de inclusión. Se identificaron cuatro estudios adicionales de las listas de referencia. Se excluyeron muchos estudios por considerarse evaluaciones de costos sin un análisis de efectividad. La mayoría de los estudios incluidos eran análisis de intervención única. Las intervenciones de atención de emergencia evaluadas por los estudios incluidos abarcaron servicios prehospitalarios, formación de proveedores, intervenciones de tratamiento, herramientas de diagnóstico de emergencia e instalaciones y paquetes de atención. La calidad de la información de los estudios era variada.

**Conclusión:**

Se encontraron grandes diferencias en las evidencias sobre la rentabilidad de las intervenciones de atención de emergencia en lugares de ingresos bajos y medios. Dada la amplitud de las intervenciones actualmente en práctica, muchas intervenciones permanecen sin evaluar, lo que sugiere la necesidad de investigaciones futuras para contribuir a las decisiones de asignación de recursos. En particular, los paquetes de intervenciones múltiples y los cambios a nivel de sistema representan un aspecto prioritario de las investigaciones futuras.

## ملخص

الغرض القيام بمراجعة منهجية وتقييم لجودة تحليلات فعالية التكلفة الخاصة بتدخلات الرعاية في حالات الطوارئ في البلدان المنخفضة والمتوسطة الدخل.

الطريقة وفقًا لإرشادات PRISMA، قمنا بإجراء بحث منهجي في كل من PubMed®، وScopus، وEMBASE®، وCochrane Library، وWeb of Science، عن الدراسات المنشورة قبل مايو/أيار 2019. وكانت معايير الدمج هي: (1) تحليل فعالية التكلفة الأصلي للتدخل، أو حزمة التدخل، في الرعاية في حالات الطوارئ، و(2) أن يكون التحليل قد حدث في بيئة منخفضة الدخل ومتوسطة الدخل. لتحديد الدراسات الأولية الإضافية، قمنا بالبحث اليدوي في قوائم مراجع الدراسات المشمولة. قمنا باستخدام دليل معايير الإبلاغ عن التقييم الاقتصادي الصحي الموحد لتقييم جودة الدراسات المشمولة.

النتائج من بين المقالات التي حصلنا عليها والتي وصل عددها إلى 1674 مقالة، حققت 35 مقالة معايير الاشتمال. لقد حددنا أربع دراسات إضافية من قوائم المراجع. واستبعدنا العديد من الدراسات التي تم اعتبارها ذات تقييمات مكلفة، دون تحليل الفعالية. وكانت معظم الدراسات المشمولة تحليلات لتدخل واحد. إن تدخلات الرعاية في حالات الطوارئ، والتي تم تقييمها بواسطة الدراسات المشمولة، غطت خدمات ما قبل المستشفى، وتدريب مقدمي الخدمات، وتدخلات العلاج، وأدوات ومرافق تشخيص الطوارئ، وحزم الرعاية. وكانت جودة تقارير الدراسات متنوعة.

الاستنتاج لقد وجدنا ثغرات كبيرة في الأدلة المتعلقة بفعالية تكلفة تدخلات الرعاية في حالات الطوارئ، في البيئات ذات الدخل المنخفض وذات الدخل المتوسط. وفي ظل اتساع نطاق التدخلات الحالية في الممارسة العملية، تظل العديد من التدخلات دون تقييم، مما يشير إلى الحاجة إلى أبحاث مستقبلية للمساعدة في قرارات تخصيص الموارد. على وجه التحديد، تمثل حزم التدخلات المتعددة والتغييرات على مستوى النظام مجالًا له الأولوية في أبحاث المستقبل.

## 摘要

**目的:**

旨在系统地评审和评估中低收入国家紧急医疗救援干预成本效益分析的质量。

**方法:**

我们遵循 PRISMA 指南，系统地搜索了 PubMed®、Scopus、EMBASE®、考克兰 (Cochrane) 图书库和 Web of Science，以查找 2019 年 5 月之前发表的研究。纳入标准为：(i) 紧急医疗救援干预或干预配套方案的原始成本效益分析，以及 (ii) 在中低收入环境中进行的分析。为了确定其他主要研究，我们对纳入研究的参考文献列表进行了手工检索。我们采用《综合健康经济评估报告标准》(Consolidated Health Economic Evaluation Reporting Standards) 指南来评估纳入研究的质量。

**结果:**

在我们确定的 1674 篇文章中，有 35 篇符合纳入标准。我们从参考文献列表中确定了另外四项研究。由于多项被视为成本评估的研究并没有进行效益分析，因此我们将其排除在外。纳入最多的研究是单项干预分析。通过纳入研究评估的紧急医疗救援干预包括院前服务、医护人员培训、治疗干预、紧急诊断工具以及设施与护理配套方案。研究的报告质量各不相同。

**结论:**

就中低收入环境中的紧急医疗救援干预而言，我们发现成本效益方面的证据差距很大。鉴于目前所实施干预的广泛程度，许多干预仍未得到评估，这表明需要进行进一步的研究以帮助做出资源分配决策。尤其是，多种干预配套方案和系统层面变更体现出未来研究的优先领域。

## Резюме

**Цель:**

Провести систематический обзор и дать оценку качеству анализа экономической эффективности мероприятий неотложной помощи в странах с низким и средним уровнем дохода.

**Методы:**

В соответствии с рекомендациями PRISMA авторы провели систематический поиск по базам данных PubMed®, Scopus, EMBASE®, Cochrane Library и Web of Science для выявления публикаций, датируемых сроками до мая 2019 г. В обзор были включены данные, выбранные по следующим критериям: (і) исходный анализ экономической эффективности мероприятий (или комплексов мероприятий) неотложной помощи; (ii) анализ проводился в странах с низким и средним уровнем дохода. Для выявления дополнительных исходных исследований авторы провели поиск также по спискам литературы в тех исследованиях, которые были ими использованы при составлении обзора. Для оценки качества включенных исследований использовались рекомендации стандартов консолидированной отчетности о состоянии экономики в здравоохранении.

**Результаты:**

Всего было выявлено 1674 статьи, из которых критериям включения отвечали 35. Авторы нашли еще 4 дополнительных исследования, изучив списки литературы. Многие исследования не вошли в обзор, так как они содержали оценку затрат, но не анализ их эффективности. Большинство вошедших в обзор исследований представляли собой анализ одного мероприятия. В изученных исследованиях рассматривались такие мероприятия неотложной помощи, как добольничные услуги, обучение поставщиков услуг, лечебные мероприятия, средства неотложной диагностики, а также объекты медицинской помощи и комплексная помощь. Качество отчетности в упомянутых исследованиях было неоднородным.

**Вывод:**

Авторы обнаружили значительные недочеты в доказательствах, применяющихся в оценке экономической эффективности мероприятий неотложной помощи в странах с низким и средним уровнем дохода. С учетом широкого диапазона мероприятий, существующих в сегодняшней практике, многие из них остаются без надлежащей оценки, что говорит о необходимости дальнейших исследований, которые помогли бы правильно принимать решения о выделении средств. В частности, в первую очередь нужно проводить такие исследования в области комплексных мероприятий и системных изменений.

## Introduction

Emergency care is a health systems and service delivery innovation that facilitates early recognition and life-saving interventions for time sensitive acute injuries and illnesses, where a delay of hours may result in avoidable death or disability, or make treatments less effective.^1^^,^^2^ Frontline providers deliver these interventions across the emergency care system, from scene care to transport to facilities. Conditions addressed by emergency care include trauma, infections, noncommunicable disease and complications of pregnancy. These conditions accounted for nine of the 10 leading causes of death in low-income countries in 2017.^3^ For people aged 5–29 years, the most common cause of death is road traffic crashes, causing over 28 million deaths a year, of which most occurring in low- and middle-income countries.^4^ Researchers have estimated that over half of deaths in low- and middle-income countries, and up to 2.5 billion disability-adjusted life-years (DALYs) annually, could be addressed through the implementation of effective emergency care.^2^ These figures are expected to grow due to factors such as increased use of motor vehicles, increased urbanization and lifestyle changes leading to increases in coronary heart disease. Traumatic injury alone is anticipated to represent a fifth of all ill-health worldwide by 2020.^5^^,^^6^ Early recognition of acute conditions by the health-care system, and improved access to care, could address much of the ill-health burden and save millions of lives. These facts have been acknowledged by the World Health Assembly Resolution *Emergency care systems for universal health coverage: ensuring timely care for the acutely ill and injured*.^7^

Emergencies occur regardless of whether a health system is prepared to address them. An organized emergency care system can theoretically leverage economies of scope and scale by employing simple low-cost interventions that will save millions of lives. However, little is known about the cost–effectiveness of emergency care interventions in low- and middle-income countries (LMIC), where such interventions may have the greatest impact.

Cost–effectiveness data is essential to inform the resource allocation decisions of policy-makers and regulators. Such data encourages the prioritization of systems and interventions most likely to provide a health and economic benefit. A survey of policy-makers in Brazil, Cuba, Nepal, Norway, and Uganda demonstrated a majority preference for efficiency arguments, such as cost–effectiveness, in formalizing the health priority setting process.^8^

Here, we present a systematic review on the cost–effectiveness of emergency care interventions in low- and middle- income countries. Our aim was to characterize the existing knowledge regarding the costs and benefits of delivering emergency care in these settings, to examine the quality of cost–effectiveness analyses and to provide guidance for future research efforts.

## Methods

### Search strategy

We systematically reviewed the literature on emergency care interventions in low- and middle-income countries. We searched for peer-reviewed articles published before May 2019, in PubMed®, Scopus, Embase®, Cochrane Library and Web of Science. To capture the heterogeneity of emergency care interventions, we included components of both pre-hospital and facility-based emergency care system. An example of the search terms used is shown in Box 1 and the full search strategy is available in the data repository.^10^ We applied no language restrictions. To limit search results to our context of interest we applied the Cochrane LMIC filter.^9^ The study was designed in accordance with the Preferred Reporting Items for Systematic Review and Meta-Analyses guidelines^11^ and was prospectively registered with PROSPERO (CRD42018080145).

Box 1 **Example search terms for the systematic review on cost–effectiveness analyses for emergency care**

Example of search terms for PubMed:

(“Cost-Benefit Analysis”[mh] OR “cost benefit” OR “cost-benefit” OR “cost effective” OR “cost-effective” OR “cost effectiveness” OR “cost–effectiveness” OR “cost utility” OR “cost-utility”)

AND

(“Emergency Service, Hospital”[mh] OR “Emergency Medicine”[mh] OR “Emergency medicine”[TW] OR “Emergency services”[TW] OR “Emergency department”[TW] OR “Emergency service”[TW] OR “Emergency departments”[TW] OR “Emergency room”[TW] OR “Emergency rooms”[TW] OR “Emergency ward”[TW] OR “Emergency Unit”[TW] OR “Trauma Centers”[mh] OR “Trauma Center”[TW] OR “Trauma Centers”[TW] OR “emergency health service”[TW] OR “emergency health services”[TW] OR “emergency medical services”[TW] OR “emergency medical service”[TW] OR “accident and emergency”[TW] OR “accident & emergency”[TW] OR “a&e”[TW] OR “A & E”[TW] OR “prehospital”[TW] OR “ambulance”[TW])

AND

Cochrane Filter

Note: We used the LMIC Cochrane filter.^9^

### Selection of studies

We uploaded all identified studies into the software Covidence (Covidence, Melbourne, Australia) for review. Studies were considered for inclusion if they: (i) described a system-wide or individual emergency care intervention; (ii) were implemented in a low- and middle- income country (according to 2018 World Bank classification) or analysed costing data from a low- and middle- income country; and (iii) undertook a full economic evaluation (either a cost–effectiveness analysis, cost-utility analysis or cost-benefit analysis). We defined emergency care interventions as interventions that provide or facilitate the early care of acutely injured and ill patients, whether outside or inside a health-care facility. This definition included early critical care or surgical interventions that commonly take place in an emergency department.

Two reviewers independently assessed titles and abstracts for inclusion of the articles for the full text review. The reviewers only selected studies for full text review if both agreed that the studies met the inclusion criteria. Studies then underwent full text reviews for eligibility by two independent reviewers. Disagreements were resolved by consensus within the study team. To identify additional primary studies, we hand searched the reference lists of included studies.

### Quality assessment

Two reviewers appraised the included studies using the Consolidated Health Economic Evaluation Reporting Standards (CHEERS) checklist.^12^ To better understand the quality of data, we did a comprehensive scoring of studies, by giving one point for each fulfilled item on the checklist, however, we did not exclude any studies based on quality.

### Data abstraction

The following information was extracted from included studies: country; year of publication; intervention; comparator; time horizon; discount rate, study perspective, health outcome, sensitivity analyses and findings. We converted cost results or the cost part of the incremental cost–effectiveness ratios to 2019 Untied States dollars (US$) for comparability. Due to the lack of consensus surrounding the use of cost–effectiveness thresholds,^13^^,^^14^ we did not apply a global benchmark to each study’s results, but left the results to be interpreted within the specific study context.

## Results

### Overview of included studies

By searching the five databases, we identified 1674 unique articles. After screening titles and abstracts for eligibility, 137 articles remained for full text screening. Of these studies, 35 studies met all the inclusion criteria and were eligible for data abstraction. Additionally, we included four eligible studies identified through hand searching of reference lists (Table 1).^15^^–^^53^

**Table 1:** Findings of included studies in the systematic review on cost–effectiveness analyses for emergency care

Author, year	Country	Study type and perspective	Sample size	Intervention	Findings^a^	CHEERS score^b^
**Prehospital services**						
Hauswald et al., 1997^27^	Malaysia	Modelling, NA	NA	Establishing an emergency medical services system responding to out-of-hospital cardiac arrest	U$ 568 642 per life saved	6
Somigliana et al., 2011^23^	Uganda	Observational, district health provider	92	Implementing an ambulance service for reproductive health in a remote setting	US$ 17.97 per year of life saved	13
Jaldell et al., 2014^19^	Thailand	Modelling, NA	NA	Decreasing emergency medical services response time by 1 minute, nationally	Savings of US$ 425 million to US$ 850 million for the national health system	12
de Ramirez et al., 2014^16^	Uganda	Observational, NA	207	Establishing an emergency medical services response system	US$ 97.10 per life saved	9
Accorsi et al., 2017^15^	Ethiopia	Observational, district health provider	111	Establishing ambulance service dedicated to emergency obstetric care	US$ 27 per life year saved	17
**Provider training**						
Arreola-Risa et al., 2000^38^	Mexico	Observational, NA	866	Course on prehospital trauma life support and increased number of ambulance dispatch centres	Increased use of prehospital interventions, decreased percentage of patients who died in transport, and costed 15.9% (US$ 77 600/ US$ 488 000) of ambulance budget	9
Arreola-Risa et al., 2004^37^	Mexico	Observational, NA	866	Basic trauma training for ambulance personnel and to improve ambulance response time	For a cost of US$ 123 555, prehospital mortality declined after medic arrival on scene from 8.2% (29/353) to 4.7% (23/491)	12
Jayaraman et al., 2009^31^	Uganda	Cross-sectional, NA	307	Trauma course for lay first-responders	US$ 30–89 per life year saved	14
Carlson et al., 2012^39^	Haiti	Modelling, NA	NA	2-year orthopaedic trauma residency	Average of US$ 149 (SD: 39) per DALY averted for the health system	17
Clark et al., 2012^25^	Sierra Leone	Observational cohort study, NA	3584	Emergency triage assessment and treatment training, triage implementation, and designation of space for emergency department	US$ 165 per paediatric death averted	14
Willcox et al., 2017^33^	Ghana	Cohort study, NA	105 850	Training nurses and midwives in basic emergency obstetric and newborn care	US$ 57.34 per DALY averted for the health provider	22
**Treatment interventions**						
Jha et al., 1998^24^	Guinea	Modelling, health-care system	NA	Various treatment interventions provided for severe conditions at first level referral hospitals	Costs for per life year saved: pneumonia in children US$ 54; malnutrition US$ 73; injury US$ 483; diarrhoea US$ 129; and malaria US$ 151	16
Patel et al., 2003^51^	India	RCT, patient and government health-care provider	200	Treating acute diarrhoea in children with zinc and copper	US$ 23 per treatment of episode	18
Gregorio et al., 2007^53^	Philippines	RCT, societal	117	Zinc supplement for children with acute diarrhoea	Savings for society of US$ 3.33 for each day that diarrhoea is averted fewer than 4 days from consult, with a spending of US$ 0.04 for each case of diarrhoea lasting fewer than 4 days from consult	14
Ozelo et al., 2007^52^	Brazil	Observational, Brazilian national health service	103	rFVIIa as first-line treatment for mild-to-moderate bleeding in patients with hemophilia compared to activated prothrombin complex concentrate	When used as first-line treatment in patients with hemophilia, rFVIIa was more effective and less expensive per bleeding episode (100%; 36/36 patients; US$ 7 490) than activated prothrombin complex concentrate (56.7%; 38/67 patients; US$ 13 500)	15
Duke et al., 2008^36^	Papua New Guinea	Cohort. NA	11 291	Improved oxygen system, including pulse oximeters, supplies and protocols, for children with pneumonia	Decreased risk of death by 35% (from 4.97% to 3.22%), costing US$ 66 per DALY averted or US$ 2 205 per life saved	15
Turhan et al., 2009^32^	Turkey	Cohort, NA	290	Non-operative management of acute appendicitis	US$ 580–731 per patient treated	6
Guerriero et al., 2011^45^	India, United Kingdom and United Republic of Tanzania	Modelling, health service	NA	Tranexamic acid injection for bleeding trauma patients within 3 hours of injury	Incremental cost per life year gained was US $79 in India, US$ 76 in United Kingdom and US$ 57 in United Republic of Tanzania	22
Chen et al., 2014^48^	Malawi	Non-RCT, health-care system	87	Bubble continuous positive airway pressure for neonates in respiratory distress	US$ 55 per life year gained for the health-care system	18
Champunot et al., 2014^46^	Thailand	Observational, health-care provider	1048	Resuscitation in the emergency department and early intensive care unit admission for severe sepsis or septic shock	US$ 1 671 per life saved	17
Assuncao et al., 2014^35^	Brazil	Cohort, NA	414	Standardized protocol for severe sepsis	Mortality reduced from 57% (182/322) to 38% (35/92). Reduction of intensive care unit costs from U$ 162 005 (SD: 237 221) to US$ 100 181 (SD: 149 388) and an average gain of 3.2 life-years after discharge	14
Wang et al., 2014^42^	China	Modelling, societal	NA	Aspirin, statin, β-blocker, ACE inhibitor, ARB and heparin for non-ST-elevation myocardial infarction. For ST-elevation myocardial infarction percutaneous coronary intervention in tertiary hospitals and streptokinase in secondary hospital	Non-ST-elevation myocardial infarction: US$ 3 291 per QALY saved; ST-elevation myocardial infarction: US$ 13 054 per QALY saved	22
Castro Jaramillo et al., 2016^43^	Colombia	Modelling, health system	NA	Factor VIII treatment following a significant bleeding in patients with hemophilia A	US$ 60 557 per QALY gained	23
Irazuzta et al., 2016^30^	Paraguay	Randomized open-label study, NA	38	High dose prolonged magnesium sulfate infusion for severe asthma	Cost per treatment US$ 761–1014. Treatment expedites discharge, which results in cost saving due to reduced duration of hospital stay	8
Pinto et al., 2016^22^	Brazil	Meta-analysis and modelling, NA	NA	Tranexamic acid injection in trauma patients	US$ 17 per life year saved	12
Dayananda et al., 2017^26^	South Africa	Cohort, NA	501	Selective non-operative management of penetrating abdominal trauma	Compared to mandatory laparotomy, intervention is effective (all patients treated survived with no complications) and saves US$ 197 263 for the health-care provider	11
Kortz et al., 2017^40^	Malawi	Modelling, government hospital	NA	Bubble continuous positive airway pressure for paediatric severe pneumonia	US$ 14 per DALY averted	22
Dwommoh et al., 2018^50^	South Africa	RCT, patient and provider	332	Motivational interviewing and problem-solving therapy interventions to reduce substance use disorder and depressive symptoms	US$ 4–20 per patient yielded improvement in mental health measured by a per unit reduction of scores on the Alcohol, Smoking and Substance Use Involvement Screening Test and the Centre for Epidemiological Studies Depression Scale	18
Yang et al., 2018^34^	China	Observational, NA	1189	Standardized treatment for acute stroke	Standardized treatment for acute stroke dominated usual care. Saving of US$ 3.34–18.30 per 1% increment of the effective management rate	9
Tigabu et al., 2019^47^	Islamic Republic of Iran	Modelling, health-care payer	NA	Treatment of severe sepsis and septic shock	US$ 11 344–11 898 per life year gained	15
**Emergency diagnostic tools**						
Schulman-Marcus et al., 2010^44^	India	Modelling, societal	NA	Electrocardiography for patients with acute chest pain presenting to a general physician	US$ 16 per QALY gained	24
Bogavac-Stanojević et al., 2013^49^	Serbia	Observational, third-party payer	192	D-dimer testing for deep vein thrombosis	Using diagnostic VIDAS® D-dimer exclusion II assay versus Hemosil D-dimer HS assay costs US$ 0.30 versus US$ 1.58 per one additional deep vein thrombosis positive patient (without pre-test probability score), and US$ 0.72 vs US$ 1.19 per one deep vein thrombosis positive patient (with pre-test probability score) selected for compression ultrasonography	18
**Facilities and packages of care**						
Horton & Claquin, 1983^28^	Bangladesh	Modelling, NA	11 509	Comparing three services for the treatment of diarrhoea, including large hospital centre, an ambulance system and a stand-alone diarrhoeal treatment centre	Cost per death averted: large centre US$ 4 032 (SD: 1 116) if patient came by ambulance compared to US$ 589 at diarrhoeal treatment centre without ambulance	11
McCord & Chowdhury, 2003^21^	Bangladesh	Observational, NA	555	Acute care facility providing a package of emergency services, including early access to surgical and obstetric care	US$ 18 per DALY for the hospital site	13
Hu et al., 2007^29^	Mexico	Modelling, NA	NA	Increasing access to comprehensive emergency obstetric care and increasing coverage levels in the WHO Mother Baby Package standard of care	Access: US$ 380 per DALY averted; coverage: US$ 697 per life year saved and US$ 494 per DALY averted	20
Gosselin et al., 2008^18^	Cambodia	Observational, NA	957	A district trauma hospital serving as a surgical care centre for injured patients	US$ 98 per DALY averted for the health centre because of surgery care for trauma	14
Gosselin et al., 2010^17^	Haiti and Nigeria	Observational, NA	6746	Emergency surgical and trauma care facilities supported by Médecins Sans Frontières	US$ 265 in Haiti and US $204 in Nigeria per DALY averted for the health centre because of the existence of surgical trauma programmes	12
Barasa et al., 2012^41^	Kenya	Cluster RCT, health-care provider	11 314	Full implementation of emergency triage assessment and treatment guidelines	US$ 0.94 per child admitted achieving one percentage point improvement in quality measure US$ 47.41–474.44 per DALY averted for national scale up	23
Kotagal et al., 2014^20^	122 low- and middle-income countries and 44 high-income countries	Modelling, NA	6640 million	Reducing injury mortality rates in low and middle-income countries to high-income rates	2 117 500 lives could be saved per year with economic benefit ranges from US$ 245 billion–261 billion (using a human capital approach) and US$758 billion–786 billion per year (using a statistical life approach)	17

ACE: angiotensin-converting-enzyme; ARB: angiotensin-II receptor blocker; β blocker: Beta blocker; CHEERS: Consolidated Health Economic Evaluation Reporting Standards; DALY: disability-adjusted life-years; HS assay: High Sensitive assay; NA: not applicable; rFVIIa: recombinant activated factor VII; RCT: randomized controlled trial; SD: standard deviation; QALY: quality adjusted life year; US$: United States dollars.

^a^ We adjusted findings to 2019 US$.

^b^ Maximum score is 24. Details about the scoring is available in the data repository.^10^

**Fig. 1:**
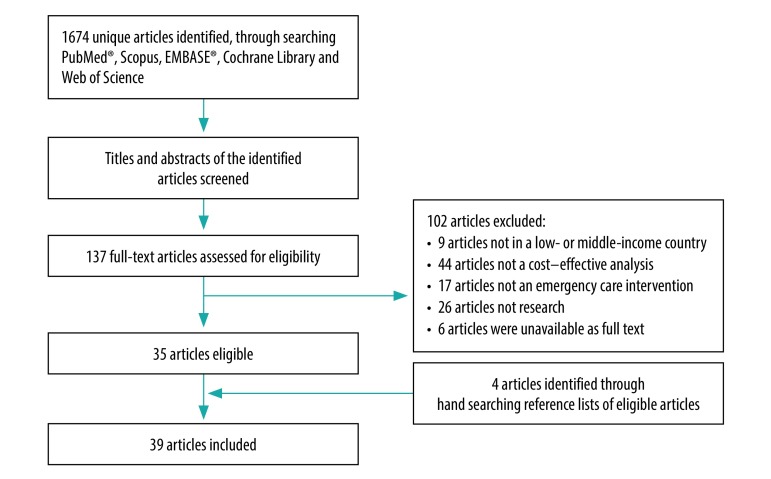
Flowchart on the selection of studies for the systematic review on cost–effectiveness analyses for emergency care interventions

The reasons for exclusion during the full text screening were: not done in a low- and middle- income country (nine studies): did not contain cost–effectiveness analyses (44 studies); did not address emergency care interventions (17 studies); were not a research article (26 studies); or were not available in full text either online or by request (six studies; Fig. 1).^10^

### Quality assessment

Table 2 shows the overall grading of quality for each article section, the detailed CHEERS scoring of each included article is available in the data repository.^10^

**Table 2:** Quality score of included studies on cost–effectiveness analyses for emergency care

Section, item	Adequately reported in study, no. (%)	Overall quality
**Title**	31 (79)	Medium
**Abstract**	37 (95)	High
**Introduction**		
Background and objectives	38 (97)	High
**Methods**		
Target population and subgroups	37 (95)	High
Setting and Location	36 (92)	High
Study perspective	17 (44)	Low
Comparators	28 (72)	Medium
Time horizon	12 (31)	Low
Discount rate	17 (44)	Low
Choice of health outcomes	29 (74)	Medium
Effectiveness	31 (79)	Medium
Preference valuation	14 (36)	Low
Costs	33 (85)	High
Currency, price date, conversion	30 (77)	Medium
Choice of model	16 (41)	Low
Assumptions	16 (41)	Low
Analytical methods	14 (36)	Low
**Results**		
Study parameters	17 (44)	Low
Incremental costs and outcomes	27 (69)	Medium
Uncertainty	17 (44)	Low
Heterogeneity	5 (13)	Low
**Discussion**		
Study findings, limitations and generalizability	37 (95)	High
**Other**		
Source of funding	23 (59)	Medium
Conflict of interest	21 (54)	Medium

Notes: We used the Consolidated Health Economic Evaluation Reporting Standards checklist.^12^ We deemed the overall quality of each item as low if the percentage was below 50%, medium if the percentage was between 50% and 80%, and high if the percentage was above 80%.

Only three studies considered a broader societal perspective,^42.42,^^53^ while 14 studies considered a health-care perspective.^15^^,^^23^^,^^24^^,^^40^^,^^41^^,^^43^^,^^45^^–^^52^ The remaining 22 studies did not clearly indicate the perspective used in the study.^16^^–^^22^^,^^25^^–^^39^ Ten studies did not clearly describe the comparator being used.^15^^–^^24^ Standardized metrics for health outcomes that allow for cross-intervention comparison were used in 26 studies. Most studies (13) used the life years saved or gained as health outcome,^15^^,^^16^^,^^20^^,^^22^^–^^24^^,^^27^^,^^28^^,^^31^^,^^35^^,^^45^^–^^48^ nine studies reported DALYs^17^^,^^18^^,^^21^^,^^29^^,^^33^^,^^36^^,^^39^^–^^41^ and three studies reported quality adjusted life years (QALYs).^42^^–^^44^ The other 13 studies reported findings in unique metrics that prohibit comparison to other disease programmes.^19^^,^^25^^,^^26^^,^^30^^,^^32^^,^^34^^,^^37^^,^^38^^,^^49^^–^^53^

Only 12 studies clearly stated the structural assumptions underpinning their decision-analytic model^19^^,^^20^^,^^29^^,^^33^^,^^39^^–^^45^^,^^49^ and only 15 studies adequately described the analytical methods used to support their evaluations.^20^^,^^24^^,^^25^^,^^29^^,^^33^^,^^36^^,^^40^^–^^44^^,^^48^^,^^49^^,^^51^^,^^53^

Although all studies analysed both costs and health outcomes, 12 studies failed to report an incremental cost–effectiveness ratio.^19^^,^^20^^,^^26^^,^^27^^,^^30^^,^^32^^,^^34^^,^^37^^,^^38^^,^^48^^,^^51^^,^^52^

Of the 17 studies reporting uncertainty analyses, 10 were deterministic^15^^,^^33^^,^^39^^,^^41^^–^^44^^,^^47^^,^^50^^,^^51^ and seven used probabilistic sensitivity analyses.^23^^,^^29^^,^^31^^,^^40^^,^^45^^,^^49^^,^^52^ The remaining 22 studies did not adequately report the use of sensitivity analyses to assess uncertainty of either parameters or the model.^16^^–^^22^^,^^24^^–^^28^^,^^30^^,^^32^^,^^34^^–^^38^^,^^46^^,^^48^^,^^53^ Only five studies reported on analysis of heterogeneity, making this item the most poorly reported checklist item.^16^^,^^18^^,^^37^^,^^44^^,^^48^

### Description of included studies

Included studies fell within five broad categories: prehospital services; training; treatment interventions; diagnostic tools; and facilities and/or packages of care. These categories are not mutually exclusive, but rather reflect the primary nature of the intervention studied. Table 1 shows a summary of all included articles, interventions assessed and main findings.^10^

#### Prehospital services

Five studies investigated the impact of either introducing a professional ambulance service or improving ambulance response times. Two studies looked specifically at the establishment of ambulance services for obstetric care,^15^^,^^23^ one at establishing a general emergency medical services system,^16^ one for cardiac arrest care,^27^ and one modelled the impacts of a decrease in response time.^19^ The cost–effectiveness of establishing an ambulance system ranged from US$ 18 in Uganda to US$ 568 642 in Malaysia per life year saved.^23^^,^^27^ In Thailand, reducing the response time would decrease the yearly national health-care expenditure by US$ 425 million to US$ 850 million for each minute saved.^19^ The wide range of results in this category is attributable to significant heterogeneity in the costs, exact interventions provided and impact data used for the analysis.

#### Provider training

Six studies assessed the impact of training interventions,^25^^,^^31^^,^^33^^,^^37^^–^^39^ three of which studied prehospital training.^31^^,^^37^^,^^38^ These prospective studies assessed training in a variety of cadres, from lay first-responders to orthopaedic specialists. Strengthening the human resource capacity to provide trauma care ranged from US$ 30 per life year saved for lay first-responders to US$ 188 per life year saved for orthopaedic trauma residency.

#### Treatment interventions

Nineteen studies compared different treatments or patient care pathways. Four studies assessed the treatment of acute bleeding, including two for tranexamic acid injection in trauma patients^22^^,^^45^ and two studies for recombinant activated factor VII injection in patients with hemophilia.^43^^,^^52^ Cost–effectiveness for tranexamic acid was between US$ 17 and US$ 76 per life year saved. For recombinant activated factor VII injection, the total direct cost was US$ 7490 per bleeding episode and US$ 60 557 per QALY gained. Other studies assessed operative versus non-operative management of appendicitis,^32^ treatment interventions for severe conditions at first level referral hospitals,^24^ treatment of abdominal penetrating trauma,^26^ severe asthma,^30^ acute myocardial infarction,^42^ paediatric respiratory distress,^36^^,^^40^^,^^48^ acute paediatric diarrhoea,^51^^,^^53^ severe sepsis and septic shock,^35^^,^^46^^,^^47^ protocolized treatment for acute stroke^34^ and substance abuse interventions.^50^

#### Emergency diagnostic tools

Two studies assessed emergency care diagnostic tools.^44^^,^^49^ For example, a modelling study showed that using an electrocardiogram for patients with chest pain in India costs US$ 16 per QALY gained.^44^

#### Facilities and packages of care

Four studies assessed the cost–effectiveness of the provision of facility-based emergency care. Three studies focused on the provision of surgical care^17^^,^^18^^,^^21^ while one evaluated a stand-alone diarrhoea treatment centre.^28^ The cost–effectiveness of facilities providing surgical care varied from US$ 18 to 265 per DALY averted,^17^^,^^18^^,^^21^ and US$ 4032 per death averted for a diarrhoeal treatment centre.^28^

Although most studies analysed actual interventions, three studies used modelling to predict the impact of increased coverage and improved quality of service.^20^^,^^29^^,^^41^ Authors of one paper estimated that the economic benefit would be within the range of U$ 758 billion–786 billion per year globally if the mortality rates in low- and middle-income countries were reduced to the rates in high-income countries.^20^ Other findings show that implementing guidelines and improving the standard of care yielded incremental cost–effectiveness ratios between US$ 47 and US$ 474 per DALY.^41^

## Discussion

We sought to systematically collect and critically appraise the existing literature on the cost–effectiveness of emergency care interventions in low- and middle- income countries. Cost–effectiveness analyses are important for assessing the value for money of emergency care interventions and to allow for prioritization and optimal resource allocation.

Formulating a general conclusion about the wider implication of the findings on the cost–effectiveness of emergency care is problematic, because of the heterogeneity of methods, settings, and presentation of results of the identified studies. For example, few studies used health outcomes that are widely comparable against other disease programmes, such as QALYs or DALYs. This lack, coupled with inconsistent reporting of incremental cost–effectiveness ratios, makes the comparison between the findings of these assessments and other programmes difficult for decision-makers with constrained budgets. Furthermore, some studies fell short of using a contextually-oriented study design and, where possible, empirically derived local inputs. For example, one study used parameters from a high-income setting to estimate cost–effectiveness in a middle-income country, generating results that are difficult to interpret.^27^

Overall, we noted that most of the studies were methodologically weak by the quality criteria we applied, failing to provide detailed descriptions of the assumptions taken. Assumptions used to calculate costs and outcomes can greatly influence the final cost estimate and reporting these details can help decision-makers understand to what level these findings apply to their setting and what level of uncertainty was taken in the review. Furthermore, not reporting the comparator used will hinder readers to understand the context of the results. Only two-thirds of the studies provided an incremental cost–effectiveness ratio, which aid decision-makers by allowing for comparability across interventions and the application of a cost–effectiveness threshold.

Even in the setting of standardized methods and results reporting, there continues to be a lack of expert consensus surrounding the interpretation of cost–effectiveness data outside the original study context that produced it. While the application of gross domestic product-based global thresholds remains a common approach, consideration of willingness-to-pay for health benefits, identification of benchmark interventions, assessment of budgetary-impact, and incorporation of league tables allow for improved contextualization of results and utility for decision-makers.^13^^,^^14^^,^^54^ When comparing the results of our included studied with readily available collated data from other public health interventions, we recommend readers go to the primary literature and ensure context and methodologic comparability.

Another notable finding from our review is that the research focused on single-intervention analyses rather than intervention packages or system changes. An organized emergency care system has the capacity to treat a variety of conditions with a common set of resources, thus gaining efficiencies in per-unit costs by applying economies of scope. Additional positive effects across the health system, such as reducing downstream health-care costs, contributing to public health surveillance and preparedness for disasters, can be also achieved by the organization and alignment of emergency care services. More research is needed on the cost–effectiveness of system changes, process improvement and intervention packages. Furthermore, an exclusive health-care perspective was used in most the studies, which may undervalue the broad social impacts and economic burden of lost workforce productivity that can be mitigated with emergency care. This narrow scope of analysis may obscure the broader productivity and economic gains that emergency care interventions provide.

Over 80 studies excluded in the review were costing-only assessments (i.e. no measure of efficacy or benefit was assessed). These studies were often descriptive costing studies of a disease entity used to justify spending on preventive measures. For example, the authors of a cost analysis of interpersonal violence in South Africa concluded that the costs of in-hospital care of violence victims warrants investment in primary prevention of these injuries.^55^ Costing-only studies were also employed for budget-impact analysis between two choices, including (i) contrasting expenditures between two health-care settings for a single disease entity^56^ and (ii) contrasting expenditures between two patient populations.^57^ One study reported an incremental cost–effectiveness ratio calculated by using the reduction in treatment time as a primary outcome rather than a health outcome and therefore was not extracted for final review.^58^

When evaluating the research gap, we noticed a sizable discrepancy between the breadth of emergency care interventions in low- and middle- income countries and the amount of published research from these settings. Of the articles we assessed, only 24 out of 137 low- and middle- income countries globally are represented in our findings, indicating a significant gap in research.

Finally, a limitation of our study surrounds the difficulty of labelling emergency care interventions for searchability. Although, we attempted to capture all literature related to emergency care, there may be relevant articles, which were not caught in our search criteria. Unless authors clearly tagged the intervention with terms related to “emergency care,” their study may not have been captured by our search. For this reason, we hand-searched references of included articles, yielding several additional studies.

Our systematic review demonstrates a relative sparsity of evidence regarding the cost–effectiveness of emergency care interventions in low-and middle-income countries. Given the breadth of available interventions, numerous potentially low- and high-cost interventions and their impacts remain unevaluated. Our review highlights areas for improvement in the quality of methods and study-design that would facilitate the use of future studies in the decision-making process with regards to the allocation of resources. Overall, the included studies allow us to begin to characterize the literature and establish a research agenda in this area. A primary focus of the future research is the development of cost–effectiveness analyses that evaluate emergency care as a system of integrated care delivery, considering economies of scope and the broader impact of organizing, and aligning health-care provision.
